# Dentist attitudes and responsibilities concerning child sexual abuse. A review and a case report

**DOI:** 10.4317/jced.52301

**Published:** 2015-07-01

**Authors:** Arturo Garrocho-Rangel, Raúl Márquez-Preciado, Ana-Isabel Olguín-Vivar, Socorro Ruiz-Rodríguez, Amaury Pozos-Guillén

**Affiliations:** 1 DDS, MS, PhD, Associate Professor, Pediatric Dentistry Postgraduated Program, Faculty of Dentistry, San Luis Potosi University, San Luis Potosí, SLP México; 2 DDS, MS, Associate Professor, Pediatric Dentistry Postgraduated Program, Faculty of Dentistry, San Luis Potosi University, San Luis Potosí, SLP México; 3 Undergraduate Student, Faculty of Dentistry, San Luis Potosi University, San Luis Potosí, SLP México

## Abstract

According to the World Health Organization, child abuse and neglect is “every kind of physical, sexual, emotional abuse, neglect or negligent treatment, commercial or other exploitation resulting in actual or potential harm to the child’s health, survival, development or dignity in the context of a relationship of responsibility, trust or power”. The aim of the present report is to inform about the most relevant aspects of child abuse and the characteristics of injuries to the head, neck, and orofacial regions, in addition to the suggested role of, and management by, the dentist for the evaluation of this condition, and also for reporting a case of a physically and sexually abused girl aged 5 years 8 months. Throughout the appointments, some type of abuse in this patient was suspected by the treating dentists at the clinic, mainly due to the initial behavior exhibited by the patient in the dental chair. Based on the clinical diagnostic an intensive preventive plan and restorative treatment was realized. The timely detection of the signs and symptoms of sexual abuse, often present in the orofacial region, place the pediatric dentist in a strategic situation, with the capacity to recognize, register, and later report those cases considered as suspect, including the dental treatment delivered and the intensive behavioral-psychological management, in order to achieve acceptation by the otherwise very anxious patient of the indicated restorative and preventive dental procedures.

** Key words:**Child abuse, dentistry, behavior management.

## Introduction

Child abuse, maltreatment, or violence represents a serious violation of the child’s basic rights and is currently considered a significant international public health problem with high prevalence among all socioeconomic, educational, ethnic, religious, and professional strata, with alarming levels of morbidity and mortality (1-3). Child abuse is defined as a persistent action of commission by a parent or parents, family members, or caregivers, which results in actual or potential harm to a child’s or adolescent’s health, development, or dignity (4,5). Some factors related with typical child abuse include financial stress, family separation, illness, substance abuse, unemployment, and overcrowded housing; perpetrators of abuse are usually relatively young (often <25 years of age) and of normal intellect (1,5); likewise, Munchausen syndrome, consisting of deliberately fabricating, inducing, or exaggerating an illness (more frequently by the mother), has also been reported (6,7). Child abuse is considered a serious concern that has gained increasing attention among pediatricians, pediatric dentists, and other health professionals. Therefore, in recent years, many countries have implemented comprehensive legislation, policies, and procedures with the purposes of protecting and safeguarding children from any type of abuse (2,8-10). According to the definition, four types of child abuse may be recognized: physical violence; sexual abuse; emotional abuse, and neglect (11-13).

Otherwise, physical violence may be classified as follows: mild (few ecchymoses and abrasions); moderate (moderate ecchymoses, burns, and a single bone fracture), and severe (large burns, multiple bone fractures, and other life-threatening injuries) (12). Consequently, abuse may make child feel worthless, seriously interfere with health, normal social, psychological development, well-being, or cause permanent emotional scars (13).

The prevalence of child abuse has been reported in several countries and reflects multifactorial social and cultural differences (3,10,14). It is more widespread than any other health problem in the world; in the U.S., around 47 of every 1,000 children are suspected victims of maltreatment (1,5) and >1,000 child fatalities in 1 year associated with abuse have been reported, whereas in Europe and Latin America, its prevalence ranges from 5‒30 cases per 1,000 children annually, depending on the social background (15). However, only 1% of cases of child abuse and neglect are reported by dentists, according to some authors (9,16). Other relevant epidemiological data on child abuse in dentistry are summarized in  Table 1 (7,17-22).

**Table 1:**
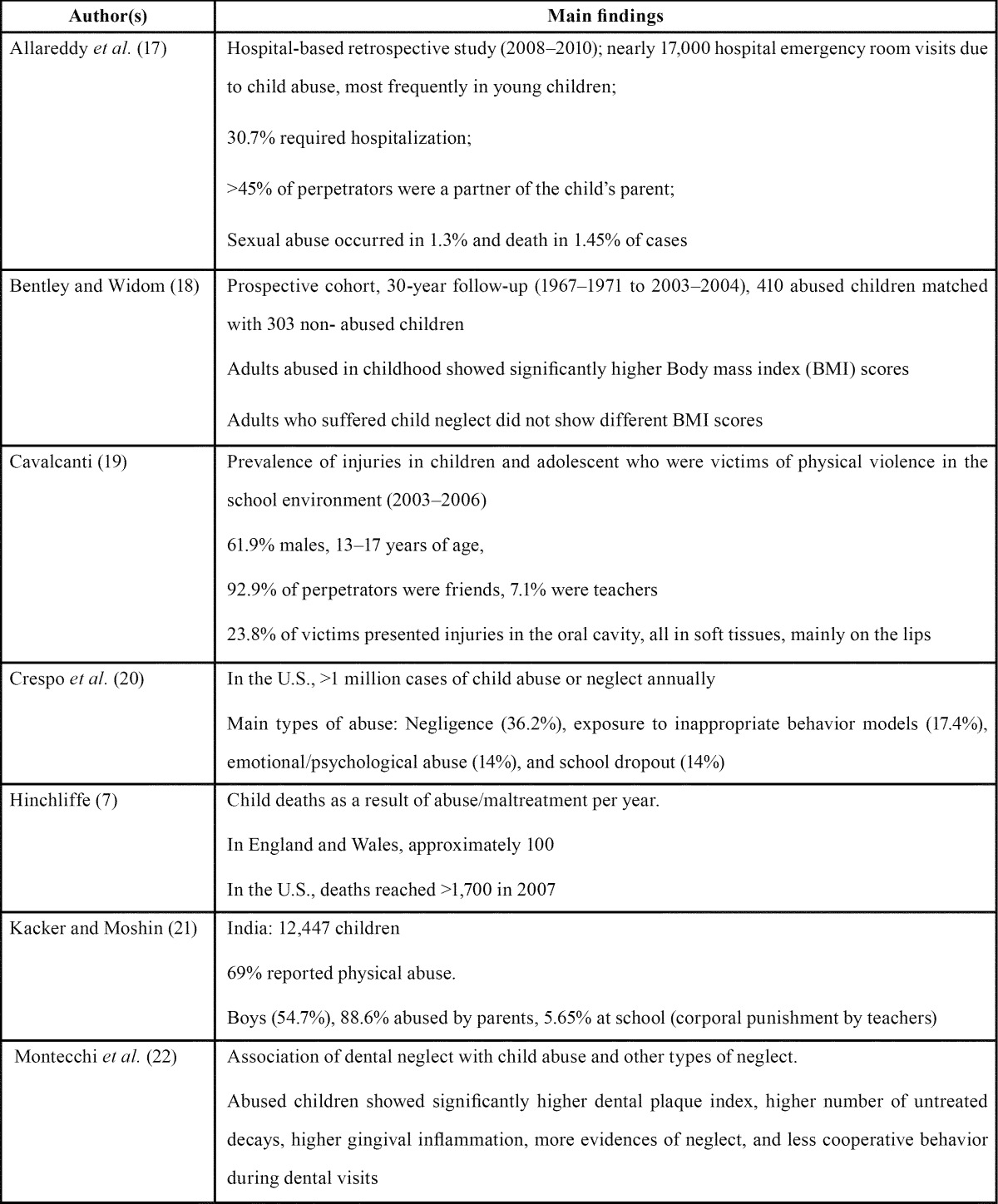
Some significant epidemiological data.

There are certain, possible general warnings and signs of child abuse, which are depicted in  Table 2. On the other hand, the majority of authors note that the head, face, and oral cavity are the central focuses for physical abuse, due to that these areas are exposed and accessible, because of their significance in nutrition and communication, and that they are representative of the whole being or self (11,12). Thus, craniofacial, head, face, and neck injuries occur in 50-75% of all cases of physical child abuse, which may be associated with other injuries elsewhere on the child’s body (23); however, Jenny *et al.* (24) revealed that 31% of infants and young children with abusive craniofacial trauma were initially misdiagnosed. Therefore, a careful and thorough intraoral and perioral assessment is always required in all cases of suspected abuse (25-27). Accidental injuries to the oral cavity, face, and head must be distinguished from those caused by abuse, by establishing whether the history, timing, and mechanism of the injury is consistent with the injury type and child’s developmental capabilities (26,28). Oral injuries may be inflicted with instruments such as bottles or eating utensils during forced feeding, by hands, fingers, belts, scalding liquids, or caustic substances, or as result of continuous behavior patterns such as whipping, biting, or severe shaking (27,29). This type of physical abuse may result in contusions, bruises, or soft tissue lacerations, most commonly on the lips, followed by the cheeks, buccal mucosa, soft and hard palate, gingivae, alveolar mucosa, and maxillary labial frenum; additionally, discolored, fractured, displaced (with pulp compromise), or avulsed teeth, facial bones, or jaw fractures are also frequent (27,30-32).

**Table 2:**
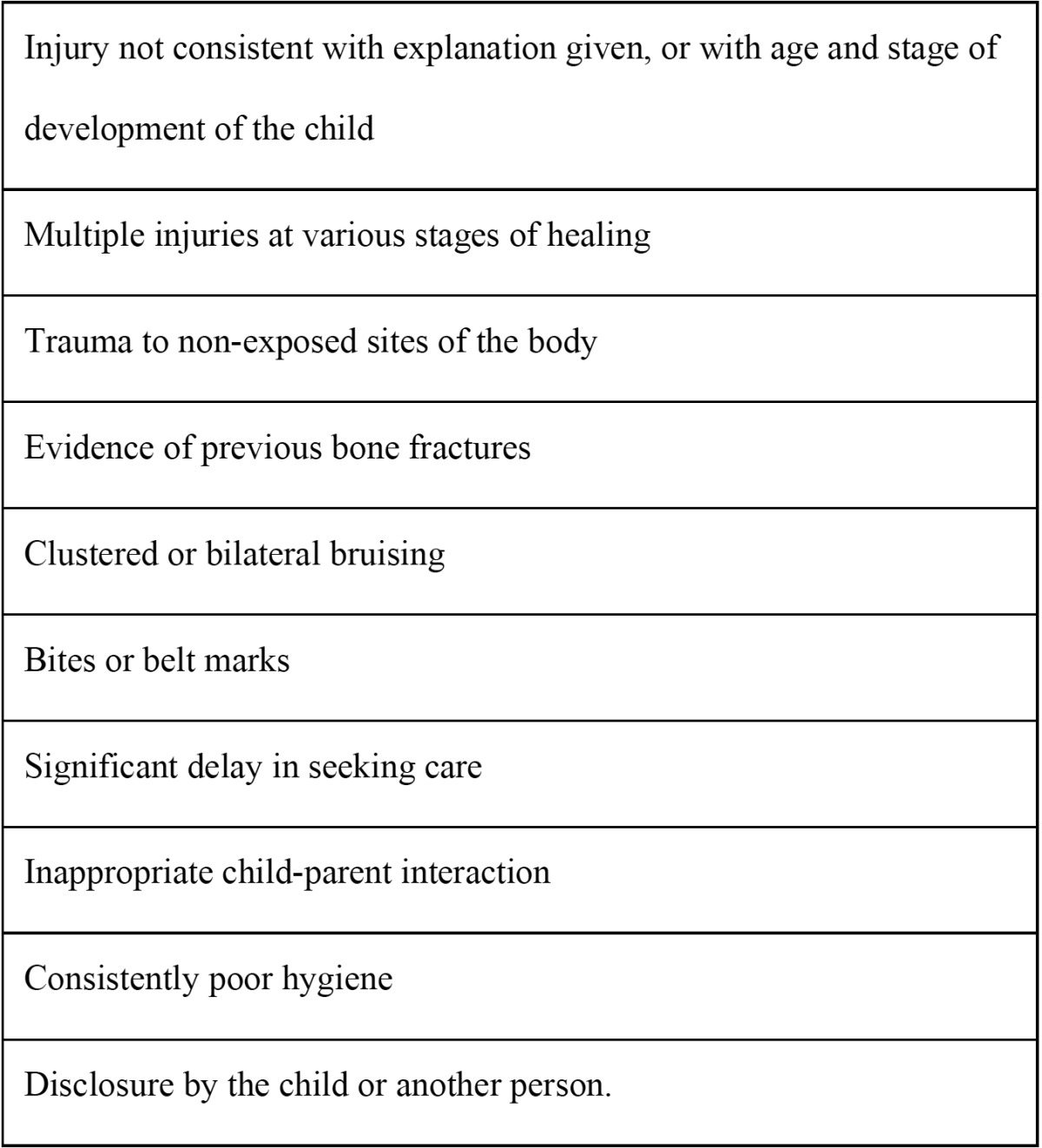
Possible general warning signs of child abuse (taken and adapted from: Hinchliffe J, 2011).

Regarding child sexual abuse, although the mouth is a frequent site for this abuse type, visible oral injuries or infections are considered rare (12,23). When oro-genital contact is suspected, as in cases of oral/perioral gonorrhea or human papillomavirus warts, referral to a specialized and well-equipped clinic setting is recommended in order to conduct comprehensive examinations (according to the AAPD, oral gonorrhea in prepubertal children is pathognomonic of sexual abuse) (10,12,33). However, other related infections may also produce oral warts. Similarly, unexplained injury or petechiae on the palate, particularly at the junction of the hard and soft palates, may be evidence of forced oral sex (27,34,35). Bite marks with ecchymoses, abrasions, or lacerations in an ovoid pattern may be also related with physical or sexual abuse (33,36,37).

## Case Report

This report describes a case of a physically and sexually abused girl aged 5 years 8 months, including the detection process and the dental treatment delivered, carried out under intensive behavioral-psychological management, in order to reach acceptance of the indicated clinical procedures by the affected patient. In October 2013, a girl aged 5 years 8 months was brought by her maternal aunt, who was also her legal guardian, to the Pediatric Dental Clinic at the Faculty of Dentistry, requesting dental evaluation and treatment, although with no complaint of pain by the patient. Based on her family history, the girl was the last-born among three siblings (she and two adolescent half-brothers) from a single mother, and attended a local preschool; medically, the patient had no adverse past history and exhibited neither systemic disease nor relevant growth and development problems. However, the girl was receiving psychological therapy at the time, and a maternal history of alcoholism and substance abuse was reported. She had not received previous dental care. According to her legal guardian, the girl exhibited an abnormal habit of introducing objects to her mouth. In addition, an introverted, silent, and fearful attitude was initially noted.

**Figure 1:**
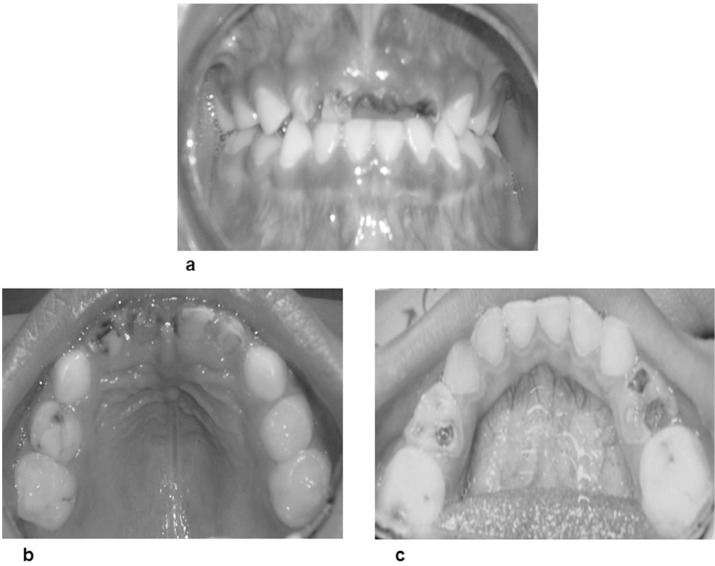
a) Frontal intraoral view; b) Intraoral view of maxillary arch; c) Intraoral view of mandibular arch.

Extraoral examination showed no evidence of skin injuries or lacerations, facial asymmetry, or temporomandibular joint abnormalities. It is noteworthy that girl’s legal guardian did not allow the taking of extraoral photographs. Intraoral examination (Fig. 1) was difficult due to the great anxiety exhibited by the patient; the most important intraoral findings were the following: complete primary dentition with several asymptomatic, deep carious cavities in the majority of her teeth, mainly in her four upper incisors, first molars (except for the upper left one), and the lower-left second molar, a condition diagnosed as early childhood caries, and as Tinanoff dental caries risk scale 5 (38). The patient also exhibited a poor oral hygiene status, manifested by heavy plaque deposits on both anterior tooth surfaces and tongue.

Based on the clinical diagnostic data and considering the patient’s great anxiety and reluctance, we decided to begin with an intensive preventive plan; then, a restorative treatment was attempted, but the child was unable to exhibit acceptable behavior (considered as Frankl Behavior Rating scale II); thus, we were required to implement diverse psychological approach techniques, such as progressive desensitization, tell-show-do, positive reinforcement, voice modulation, and gifting, among others, but aversive methods such as hand over mouth were never applied. Fortunately, these procedures paid off, and the patient’s cooperation with and attitude toward the dental environment improved noticeably. Her anxiety was reduced to a great degree over the following appointments, to the extent that the following planned restorative treatment could be successfully performed: 2 pit and fissure sealants (on both upper-left molars); 3 composite fillings (upper-right second molar and both lower second molars); 2 pulpotomies with their corresponding preformed crowns (upper-right first molar and lower-left first molar), 1 pulpectomy with its preformed crown (lower-right first molar), and 4 upper incisor extractions. Later, an upper anterior fixed esthetic prosthesis was put in place. The final patient’s attitude was rated as excellent. Likewise, oral hygiene and nutritional strategies were provided and reinforced during the dental appointments. A non-cariogenic diet lacking fruits, juices, and other soft drinks, or animal proteins, was recommended.

Throughout the appointments, some type of abuse in this patient was suspected by the treating dentists at the clinic, mainly due to the initial behavior exhibited by the patient in the dental chair during certain situations. For example, we noted that, when a male adult approached the dental chair, the patient became visibly nervous and whispered things to us, such as: “Be careful. Men are dangerous”. Additionally, she was often found staring and retracted; also, when rubber dam isolation was set in place, her eyes exhibited mild tears, although she denied any pain. After deeper and careful inquiry, the patient’s aunt finally revealed that her the aunt’ nephew had, several months previously, genitally and orally sexually abused the patient repeatedly, while her mouth was covered with the hands of her two adolescent half-brothers. She also referred that the neighbors often heard her screaming; bruises and other physical maltreatment were some of the signs noticed. This situation was previously reported to the Mexican Government Child Protective Services, custody of the child was soon legally awarded to her maternal aunt, and a psychological approach was immediately initiated.

Shortly after finishing the dental treatment, the girl immediately moved to a nearby city because of related legal issues and repeated threats by other family members. Therefore, we were not able to see the patient for the termination appointments. Unfortunately, because of this, we could neither provide our final recommendations nor take postoperative intraoral photographs. However, we were very satisfied and pleased with the outcomes obtained, in all aspects, and always acted by means of the most humane manner possible.

## Discussion

Every child worldwide has the right of a loving environment and deserves a life free from violence, and child protection is the duty of every member of the society (10,15). However, millions of children around the world are victims of and/or witness to physical, sexual, and emotional violence (4,5). As previously mentioned, the majority of the lesions reported due to child abuse involve the mouth, craniofacial regions, and neck. Therefore, pediatric dentists are in an exceptionally favorable position to recognize child abuse. Although parents who abuse their children tend to change their child’s physician frequently, they are more likely to continue visiting their child’s dentist (26). Additionally, pediatric dentists have regular contact and a relationship with the children and families; thus, they can observe the psychological condition of the patients and their family environment (7,12). Additionally, orofacial trauma in children commonly presents at pediatric dentists’ offices; thus, these dentists can identify signs of possible child physical or sexual abuse. Therefore, they should be able to recognize these signs and take the responsibility to inform themselves community procedures for child protection (8,9,14). However, many dental health professionals often un-der-report child abuse, mainly due to fear, lack of knowledge, and training, although their potential role in detecting this crime is growing (4,17).

Pediatric dentists or general dentists who are likely to treat children should be involved in aiding in the prevention of child physical or sexual abuse. Thus, they must be further trained to recognize and register some of the more common oral or perioral manifestations of this abuse, and then report any suspicion of abuse (5,7). Knowing about familial and environmental contexts where the child is growing up can lead to working out a more effective preventive effort in order to protect these vulnerable children (3,9).

Whenever suspicions of physical or child sexual maltreatment arise, a routine protocol should be followed (23). It is essential to elaborate a thorough and detailed clinical history, preferably directly from the child, that accurately documents all possible relevant explanations from parents or caregivers in a careful and prudent manner, in an attempt to contrast all of this information against the clinical findings, in order to find the truth regarding unusual oral, perioral, or behavioral manifestations (5,15,33). In cases in which sexual abuse is suspected, as in the case reported here, careful inquiry is also suggested. Thus, the dentist should question the child and the parent separately with regard to what caused any observed injury, and must also take into consideration the his/her own attitude toward a situation involving these features and provide a quiet, safe, and comfortable environment in the dental office (7,12). In many cases, the caregiver fails to provide the dentist with this information at the patient’s initial appointments, perhaps due to shame, ignorance, or self-protection: the sexual perpetrator is usually one or more close relatives, and other relatives could act as tolerating, overlapping, and even covering up the fact. Therefore, the information supplied may be purposely biased. In the present case, the legal guardian did not inform us about the patient’s sexual abuse because she considered that it was not relevant.

Multiples injuries or injuries at different healing stages, or a discrepant history should arouse the suspicion of physical and sexual abuse (23). Also, it is important to observe the parent (or caregiver)-child relationship, as well as the changes in the child’s behavior; a parent may bring a child to the dentist to have loose or broken teeth treated, but may not seek treatment for other types of injuries (7,33). All suspected events should be reported to the corresponding government services for investigation, specialized laboratory testing for semen or foreign material (only in recent cases), and for specialized multidisciplinary management (12). If the patient’s age permits it, careful direct questions may be asked, but always in the presence of witnesses, such as staff members, a social worker, or the guardian. X-rays, photographs, and impressions of the anatomical structures involved must be taken, when necessary (13). Likewise, some oral or perioral injuries can sometimes be confirmed by laboratory findings, which could provide the timing or nature of these injuries, as well as the identity of the perpetrator (5,27).

If doubt exists concerning whether to report or not the suspected case, consultation with the patient’s physician, a social worker, or with the local government authorities is recommended (9). On the other hand, when treating these children, all of dentist’s knowledge, skill, and experience must be applied in order to obtain as much cooperation as possible from the patient, by means of employing the diverse pharmacological or non-pharmacological behavior management approaches available, such as those employed in the present case, always attempting to avoid those considered as aversive, and taking into consideration the high level of anxiety or fear often exhibited by abused children (1,26,29). With respect to our patient, we suppose that the placement of rubber dam isolation caused the girl to feel threatened because the rubber dam covered her mouth, resembling the hand(s) held over her mouth while she underwent sexual abuse (12).

Child maltreatment or violence is, in the majority of cases, a traumatic experience that causes a variety of cognitive, behavioral, and emotional responses, including anxiety and depression, sleep disorders, fear, difficulties in concentration, low self-esteem, propensity to suicide, addictive habits, lack of sphincter control, and even suicide, among others (7,13). All of these health and social problems may persist into adulthood (4,8). Thus, multidisciplinary professional and legal management and long-term fo-llow-up of cases of child abuse is strongly recommended (23).

Under these circumstances, dental health personnel are encouraged to consult the expanding body of literature on the subject of child abuse, to increase their understanding of the nature and prevalence of violence toward or maltreatment of children in its different forms (1,13). The protection of a mistreated child must be our main concern. Dentists have a great opportunity to take a proactive role in helping these victims.

All over the world, adequate qualification of dentistry professionals is essential for proper clinical and behavioral management of orofacial injuries or emotional damage resulting from any type of child maltreatment. All dentists should learn and assume, as professional obligations, the identification, as early as possible, of the signs or symptoms characteristic of child abuse and neglect when confronted with a suspected case, and must become fully aware of the appropriate registration and reporting procedures. Such efforts could increase our ability to protect and care for our patients, who are often incapable of self-protection.

## Conclusions

It is necessary for dentists to collaborate among themselves to increase the prevention, detection, and treatment of this condition and the urgent adoption of protective measures for the victim, as well as adequate follow-up in each case, in order to avoid the recurrence of physical or sexual abuse in unfortunate and/or defenseless children.
